# Targeted knockdown of PGAM5 in synovial macrophages efficiently alleviates osteoarthritis

**DOI:** 10.1038/s41413-024-00318-8

**Published:** 2024-03-04

**Authors:** Yuhang Liu, Ruihan Hao, Jia Lv, Jie Yuan, Xuelei Wang, Churong Xu, Ding Ma, Zhouyi Duan, Bingjun Zhang, Liming Dai, Yiyun Cheng, Wei Lu, Xiaoling Zhang

**Affiliations:** 1 Department of Orthopedic Surgery, Xin Hua Hospital Affiliated to Shanghai Jiao Tong University School of Medicine (SJTUSM), Shanghai, 200092 China; 2 National Facility for Translational Medicine, Shanghai, 200240 China; 3 Shanghai Frontiers Science Center of Genome Editing and Cell Therapy, Shanghai Key Laboratory of Regulatory Biology, School of Life Sciences, East China Normal University, Shanghai, 200241 China; 4 Department of Orthopaedic Surgery, The Second Hospital of Shanxi Medical University, Taiyuan, 030001 China; 5 CAS Key Laboratory of Tissue Microenvironment and Tumor, Shanghai Institute of Nutrition and Health, Chinese Academy of Sciences, Shanghai, 200031 China; 6 School of Medicine, Shanghai University, Shanghai, 200444 China

**Keywords:** Physiology, Diseases, Pathogenesis

## Abstract

Osteoarthritis (OA) is a common degenerative disease worldwide and new therapeutics that target inflammation and the crosstalk between immunocytes and chondrocytes are being developed to prevent and treat OA. These attempts involve repolarizing pro-inflammatory M1 macrophages into the anti-inflammatory M2 phenotype in synovium. In this study, we found that phosphoglycerate mutase 5 (PGAM5) significantly increased in macrophages in OA synovium compared to controls based on histology of human samples and single-cell RNA sequencing results of mice models. To address the role of PGAM5 in macrophages in OA, we found conditional knockout of PGAM5 in macrophages greatly alleviated OA symptoms and promoted anabolic metabolism of chondrocytes in vitro and in vivo. Mechanistically, we found that PGAM5 enhanced M1 polarization via AKT-mTOR/p38/ERK pathways, whereas inhibited M2 polarization via STAT6-PPARγ pathway in murine bone marrow-derived macrophages. Furthermore, we found that PGAM5 directly dephosphorylated Dishevelled Segment Polarity Protein 2 (DVL2) which resulted in the inhibition of β-catenin and repolarization of M2 macrophages into M1 macrophages. Conditional knockout of both PGAM5 and β-catenin in macrophages significantly exacerbated osteoarthritis compared to PGAM5-deficient mice. Motivated by these findings, we successfully designed mannose modified fluoropolymers combined with siPGAM5 to inhibit PGAM5 specifically in synovial macrophages via intra-articular injection, which possessed desired targeting abilities of synovial macrophages and greatly attenuated murine osteoarthritis. Collectively, these findings defined a key role for PGAM5 in orchestrating macrophage polarization and provides insights into novel macrophage-targeted strategy for treating OA.

## Introduction

Osteoarthritis (OA) is the most common joint degenerative and age-related disorder and is characterized by cartilage degradation, ectopic osteophyte formation, subchondral bone remodeling, and synovial inflammation.^1,2^ Current standard of care does not provide satisfactory relief for many patients due to the complex pathophysiology of OA.^3^ One of the primary pathologies of osteoarthritis is chronic and low-grade inflammation mainly caused by joint damage and debris, which triggers the innate immune response in the early stage of OA and finally results in infiltration of immune cells and synovial hyperplasia.^4^ In OA synovium, M1 and M2 macrophages compete with each other in various pathologic conditions, which is critical for the homeostasis of OA.^5^ M1 macrophages accumulated more in the synovial membrane of experimental OA than M2 macrophages and further exacerbated the progression of OA, while M2 macrophages could induce cartilage synthesis and inhibit chondrocyte apoptosis.^5–7^ Thus, exploring the mechanisms underlying macrophage polarization and remodeling synovial macrophages is emerging as a strategy for OA intervention.

Phosphoglycerate mutase 5 (PGAM5) is a mitochondrial serine/threonine phosphatase located in the mitochondrial membrane,^8^ which acts as a critical regulator of mitochondrial metabolism and dynamics and controls a series of functions of cells. PGAM5 is vital in programmed cell necrosis by dephosphorylating Drp1, leading to mitochondrial fragmentation.^9^ PGAM5 also regulates mitophagy by recruiting the E3 ubiquitin ligase PARKIN or dephosphorylating FUNDC1 to modulate the degradation of mitochondria.^10^ In addition, PGAM5 modulates cellular senescence by regulating mitochondrial dynamics^11^ and was also shown to enhance inflammasome activation in macrophages, which has a critical role in processing of pro-IL-1β in bone marrow-derived macrophages (BMDMs).^12^ However, the role of PGAM5 in regulating synovial macrophages in OA has not been reported before.

**Fig. 1:**
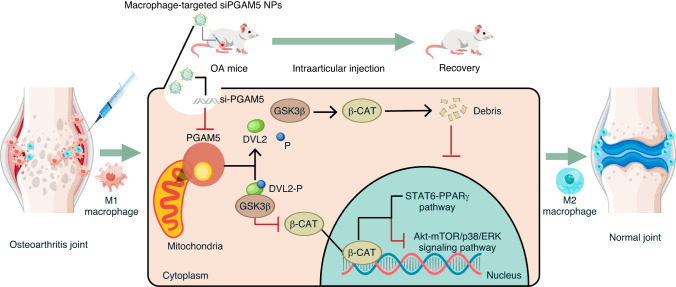
Schematic illustration of the role of PGAM5 in osteoarthritis and related macrophage-targeted therapy. PGAM5 on the mitochondrial membrane directly dephosphorylated DVL2, further led to the degradation of β-catenin via activating GSK3β, resulting in the loss of regulation of Akt-mTOR/p38/ERK and STAT6-PPARγ signals by β-catenin in nucleus, which tilted macrophages to M1 polarization. Targeted knockdown of PGAM5 in macrophages could be established via intraarticular injection of nanoparticles (NPs) composed of mannose modified fluoropolymers combined with siPGAM5, and could directly inhibited PGAM5 in synovial macrophages and greatly reduced OA symptoms in mice

We herein found macrophage PGAM5 significantly increased in macrophages in OA synovium based on histology of human samples and single-cell RNA sequencing results of mice models. Furthermore, we found that conditional knockout of PGAM5 in macrophages alleviated murine OA symptoms via repolarizing M1 macrophages into M2 macrophages in synovium. Mechanistically, we found PGAM5 enhanced M1 polarization via AKT-mTOR/P38/ERK signaling pathways, whereas inhibited M2 polarization via STAT6-PPARγ signaling pathway. Besides, we identified PGAM5 inhibited β-catenin via dephosphorylation of DVL2 to regulate macrophage polarization and conditional knockout of both PGAM5 and β-catenin in macrophages led to increased OA symptoms compared to PGAM5-deficient mice. In order to precisely modulate PGAM5 in synovial macrophages of OA, we constructed nanoparticles (NPs) composed of mannose modified fluoropolymers and siPGAM5 to inhibit PGAM5 specifically in macrophages, which significantly reduced the OA symptoms via repolarization of M1 macrophages into M2 macrophages (Fig. 1). Our results, therefore, demonstrated that PGAM5 plays a critical role in regulating macrophage polarization which has excellent potential for clinical OA treatment.

## Results

### PGAM5 expression increased in macrophages of the OA synovium

**Fig. 2:**
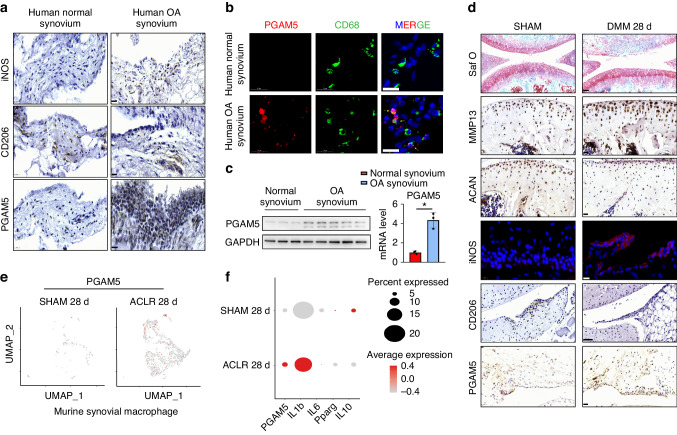
PGAM5 increased in OA macrophages in the synovium. **a** Representative immunohistochemistry images of iNOS, CD206, and PGAM5 (scale bars, 20 µm) in human normal and OA synovium. **b** Representative images of coimmunofluorescence of PGAM5 and CD68 in human normal and OA synovium. (scale bars, 20 µm). **c** WB and qPCR analysis of PGAM5 levels in human normal (*n* = 3) and OA synovium (*n* = 5). Relative protein levels with statistical analyses were listed in Fig. S4. **d** Representative images of safranin O staining, (scale bars, 50 µm), immunohistochemistry of ACAN, MMP13, PGAM5 (scale bars, 20 µm), CD206 (scale bars, 50 µm), and immunofluorescence of iNOS (scale bars, 10 µm) in knee joints 28 days after DMM surgery of WT mice (*n* = 6). **e** UMAP plot of murine synovial macrophages in sham group and 28 days after anterior cruciate ligament rupture-based model (ACLR) group and feature plots of PGAM5. **f** Expression of PGAM5, IL1b, IL6, Pparg, IL10 in clusters of murine synovial macrophages in sham group and 28 d ACLR group. Data are shown as the mean±s.d. *P < 0.05 between the indicated groups. *P* values were determined using Student’s *t* tests

To investigate the factors that modulate the progression of osteoarthritis, we examined human synovium from OA patients and normal controls (patients with femoral fracture) and observed enhanced expression of inducible nitric oxide synthase (iNOS) and reduced expression of CD206 in the OA synovium compared to controls (Fig. 2a). Immunohistochemistry showed that PGAM5 was mainly detected in the human OA synovium (Fig. 2a). To address the role of PGAM5 in OA macrophages, we further observed increased expression of PGAM5 in macrophages in human OA synovium compared to controls, as confirmed by double-positive immunostaining for PGAM5 and CD68, a marker of macrophages (Fig. 2b), indicating the potential role of PGAM5 in modulating macrophages in the OA synovium. In addition, we confirmed that the protein and RNA levels of PGAM5 increased in human OA synovium (Fig. 2c). Based on the results in human samples, we further performed destabilization of the medial meniscus (DMM) surgery in 8-week-old male wild - type (WT) mice. The knee joints of mice were collected and sectioned for histological examination 28 days after DMM surgery, and safranin O staining, immunohistochemistry of matrix metalloproteinase 13 (MMP13) and aggrecan (ACAN) in the knee joints indicated successful joint osteoarthritis induction in the DMM group (Fig. 2d). OARSI scores of safranin O staining were listed in the supplementary materials (Fig. S1). As expected, we also observed enhanced iNOS and reduced CD206 expression in the OA synovium of mice, and PGAM5 was upregulated in the murine synovium of DMM model compared to the sham group (Fig. 2d). We further investigated the database in NCBI and reanalyzed the single-cell RNA sequencing for murine synovium in an OA model induced by anterior cruciate ligament rupture^13^ and found that the expression of PGAM5 increased in the synovial cells of OA mice compared to the sham group, specifically in the macrophage cluster which showed enhanced expression of IL1b, IL6, and reduced expression of Pparg and IL10 in the surgical group (Fig. 2e, f), indicating that PGAM5 might be a potential regulatory factor of OA macrophages in the synovium.

### PGAM5 regulated osteoarthritis in mice by modulating macrophage polarization

**Fig. 3:**
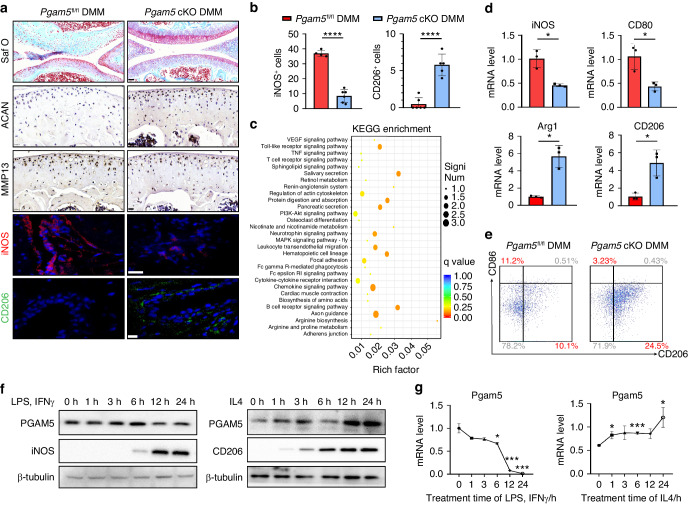
PGAM5 deletion in macrophages relieved osteoarthritis in mice by modulating polarization. Osteoarthritis was induced in *Pgam5* cKO mice and their respective control mice by DMM surgery. The knee joints of *Pgam5* cKO mice and *Pgam5*^fl/fl^ mice were analyzed 28 days after the surgery. **a** Representative images of Safranin O staining (scale bars, 50 µm), immunohistochemistry of ACAN and MMP13 (scale bars, 20 µm), and immunofluorescence of iNOS (red) (scale bars, 20 µm) and CD206 (green) (scale bars, 10 µm) in the knee joint 28 days after DMM surgery of *Pgam5* cKO mice and *Pgam5*^fl/fl^ mice (*n* = 9). **b** Quantification of immunofluorescence of iNOS (red) and CD206 (green) in the synovium of the knee joint of *Pgam5* cKO mice and *Pgam5*^fl/fl^ mice 28 days after DMM surgery. **c** KEGG analysis of BMDMs from *Pgam5* cKO mice and *Pgam5*^fl/fl^ mice 28 days after DMM surgery. **d** qPCR analysis of iNOS, CD80, Arg1, and CD206 mRNA levels in peritoneal macrophages of *Pgam5* cKO mice and *Pgam5*^fl/fl^ mice 28 days after DMM surgery. **e** Flow cytometry of peritoneal macrophages of *Pgam5* cKO mice and *Pgam5*^fl/fl^ mice 28 days after DMM surgery. **f** Western blots analysis of PGAM5, iNOS, and CD206 protein levels in BMDMs stimulated by LPS and IFNγ or IL4 for 0, 1, 3, 6, 12, and 24 h. Relative protein levels with statistical analyses were listed in Fig. S5 (*n* = 3). **g** qPCR analysis of PGAM5 mRNA level in BMDMs stimulated with LPS and IFNγ or IL4 for 0, 1, 3, 6, 12, and 24 h. One of the three independent experiments with identical results is shown. Data are shown as the mean±s.d. *P < 0.05, **P < 0.01, ***P < 0.001, ****P < 0.000 1 between the indicated groups. *P* values were determined using Student’s *t* tests

To address the role of macrophage PGAM5 in osteoarthritis, we generated *Pgam5*^fl/fl^ -lyz2-Cre (*Pgam5* cKO) mice, by crossing *Pgam5*^fl/fl^ mice with transgenic mice that carried lysozyme (Lyz2) proximal promoter - mediated Cre recombinase, which specifically ablates PGAM5 from macrophages. We then employed DMM surgery in 8-week-old male *Pgam5*^fl/fl^ and *Pgam5* cKO mice for 28 days. We found that *Pgam5* cKO mice exhibited relieved OA symptoms compared to *Pgam5*^fl/fl^ mice, indicated by enhanced safranin O staining, increased expression of ACAN, and decreased expression of MMP13 in cartilage (Fig. 3a). OARSI scores of safranin O staining were listed in the supplementary materials (Fig. S2). We further observed decreased M1 and increased M2 macrophages in the synovium of *Pgam5* cKO mice compared to *Pgam5*^fl/fl^ mice, as confirmed by immunofluorescence of iNOS and CD206 (Fig. 3a, b). RNA-seq of BMDMs from *Pgam5* cKO mice and *Pgam5*^fl/fl^ mice was performed to evaluate the modulatory role of PGAM5 deletion on macrophages at the transcriptome level. Kyoto Encyclopedia of Genes and Genomes pathway analysis showed that various macrophage polarization-associated pathways, including the Toll-like receptor signaling pathway, PI3K-Akt signaling pathway, MAPK signaling pathway, and arginine biosynthesis^14^ in BMDMs of *Pgam5* cKO mice were significantly different from that in BMDMs of *Pgam5*^fl/fl^ mice, suggesting that PGAM5 potentially modulated macrophage polarization (Fig. 3c). mRNA levels of M1 markers, iNOS and CD80, and M2 markers, arginase 1 (Arg1) and CD206, were detected in peritoneal macrophages of *Pgam5* cKO and *Pgam5*^fl/fl^ mice, which indicated that *Pgam5* cKO mice showed fewer proinflammatory phenotypes than *Pgam5*^fl/fl^ mice (Fig. 3d). These results were validated by flow cytometry of peritoneal macrophages, which indicated fewer CD86^+^ (M1) and more CD206^+^ (M2) cells in *Pgam5* cKO macrophages (Fig. 3e). To further examine the potential function of PGAM5 in macrophage polarization, we examined whether the expression of PGAM5 changed during the induction of macrophage polarization. BMDMs of WT mice were collected and induced to the M1 polarized state by lipopolysaccharide (LPS) and interferon-γ (IFNγ) or induced to the M2 state by stimulation with IL4. M1 induction significantly reduced PGAM5 mRNA and protein levels within 24 h. However, M2 induction increased PGAM5 mRNA and protein levels in macrophages within 24 h (Fig. 3f, g). The results above indicated that PGAM5 might be involved in regulating macrophage polarization to modulate OA.

### PGAM5 enhanced M1 polarization and inhibited M2 polarization of BMDMs

**Fig. 4:**
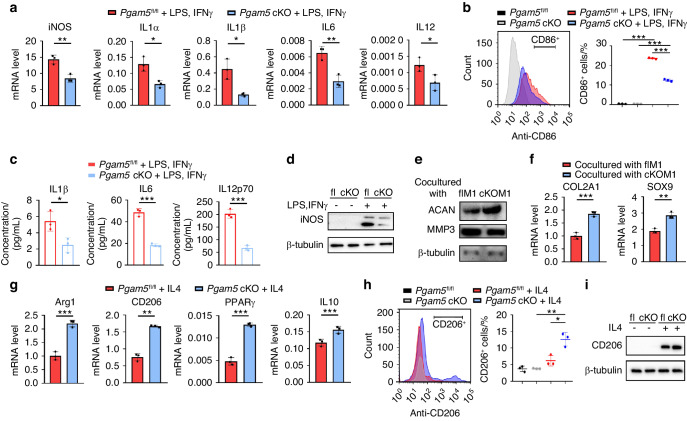
PGAM5 promoted M1 polarization, inhibits M2 polarization, and caused enhanced cartilage inflammation in vitro. Primary BMDMs of *Pgam5* cKO mice and *Pgam5*^fl/fl^ mice were freshly isolated and induced for examination. **a** BMDMs from *Pgam5* cKO mice and *Pgam5*^fl/fl^ mice were stimulated with LPS plus IFNγ for 24 h, and the mRNA levels of iNOS, IL1α, IL1β, IL6, and IL12 were determined by qPCR. **b** CD86 expression in *Pgam5* cKO and *Pgam*5^fl/fl^ macrophages stimulated by LPS plus IFNγ was determined by flow cytometry. **c** The concentrations of IL1β, IL6, and IL12p70 in the culture medium of *Pgam5* cKO and *Pgam5*^fl/fl^ BMDMs stimulated by LPS plus IFNγ for 24 h were detected by enzyme-linked immunosorbent assay. **d** Western blot analysis of iNOS protein level in BMDMs from *Pgam5* cKO and *Pgam5*^fl/fl^ mice produced by LPS plus IFNγ was determined by western blots. Relative protein levels with statistical analyses were listed in Fig. S6a (*n* = 3). **e** Freshly isolated BMDMs from *Pgam5* cKO and *Pgam5*^fl/fl^ mice were cultured and stimulated with LPS plus IFNγ for 24 h and cocultured with chondrocytes isolated from WT neonatal mice for 24 h. Protein expression of ACAN and MMP3 was determined by western blots. Relative protein levels with statistical analyses were listed in Fig. S6c, d (*n* = 3). **f** qPCR analysis of COL2A1 and SOX9 mRNA levels. **g** BMDMs from *Pgam5* cKO and *Pgam5*^fl/fl^ mice were stimulated with IL4 for 24 h, and the expression of Arg1, CD206, PPARγ, and IL10 was determined by qPCR. **h** CD206 expression in *Pgam5* cKO and *Pgam5*^fl/fl^ macrophages stimulated by IL4 was determined by flow cytometry. **i** Western blot analysis of CD206 protein level in BMDMs from *Pgam5* cKO and *Pgam5*^fl/fl^ mice produced by IL4 was determined by western blots. Relative protein levels with statistical analyses were listed in Fig. S6b (*n* = 3) Data are shown as the mean ± s.d. *P < 0.05, **P < 0.01, ***P < 0.001 compared with *Pgam5*^fl/fl^ mice or between the indicated groups. *P* values were determined using Student’s *t* tests

To further illustrate the role of PGAM5 in macrophage polarization, we isolated BMDMs from *Pgam5* cKO and *Pgam5*^fl/fl^ mice and induced them to an M1-polarized state by LPS plus IFNγ for 24 h in vitro. PGAM5 deletion in BMDMs significantly suppressed the mRNA levels of proinflammatory genes, including iNOS, IL1α, IL1β, IL6, and IL12, compared with that in *Pgam5*^fl/fl^ macrophages (Fig. 4a). The number of CD86^+^ cells was significantly lower in *Pgam5* cKO BMDMs than in *Pgam5*^fl/fl^ BMDMs after M1 induction, as determined by flow cytometry (Fig. 4b). Enzyme-linked immunosorbent assay (ELISA) of the supernatant of BMDMs treated with LPS plus IFNγ also verified that PGAM5 deletion led to decreased secretion of proinflammatory cytokines in macrophages (Fig. 4c). In addition, the protein level of iNOS was significantly lower in *Pgam5* cKO BMDMs than in *Pgam5*^fl/fl^ macrophages after M1 induction (Fig. 4d). To clarify the influence of PGAM5-deficient macrophages on cartilage, BMDMs from *Pgam5 c*KO and *Pgam5*^fl/fl^ mice were cocultured with chondrocytes after M1 induction in vitro. The protein levels of ACAN and MMP3 and the mRNA levels of COL2A1 and SOX9 in chondrocytes indicated that PGAM5 deletion in macrophages limited the proinflammatory phenotypes of chondrocytes (Fig. 4e, f).

Next, we aimed to investigate whether PGAM5 plays an essential role in M2 macrophage polarization. *Pgam5* cKO and *Pgam5*^fl/fl^ BMDMs were isolated and stimulated with IL4 for 24 h for M2 induction. After IL-4 stimulation, *Pgam5* cKO BMDMs expressed enhanced mRNA levels of Arg1, CD206, PPARγ, and IL10, compared to *Pgam5*^fl/fl^ BMDMs (Fig. 4g). The number of CD206^+^ cells was significantly higher in *Pgam5* cKO BMDMs than in *Pgam5*^fl/fl^ BMDMs after M2 induction, as determined by flow cytometry (Fig. 4h). The protein level of CD206 significantly increased in *Pgam5*^-^cKO BMDMs compared to *Pgam5*^fl/fl^ macrophages after M2 induction (Fig. 4i). In conclusion, PGAM5 led to an increased M1 response and a decreased M2 response in macrophages in vitro.

### PGAM5 induced M1 polarization via the AKT-mTOR/P38/ERK signaling pathway, whereas inhibited M2 polarization through STAT6-PPARγ signaling pathway

**Fig. 5:**
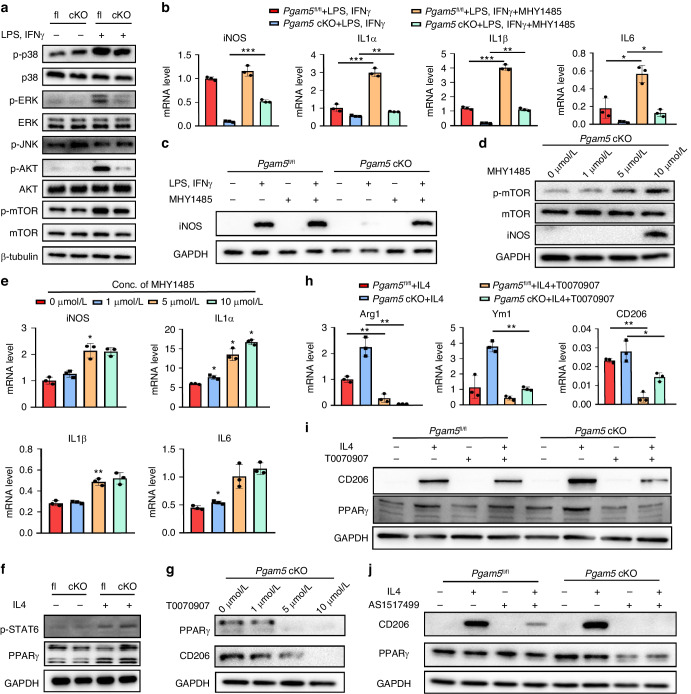
PGAM5 improved M1 polarization via Akt-mTOR/P38/ERK signaling pathway and inhibited M2 polarization via the STAT6-PPARγ signaling pathway. **a** After stimulation with LPS and IFNγ for 24 h, the protein levels of p-p38, total p38, p-ERK, total ERK, p-JNK, p-AKT, total AKT, p-mTOR, and total mTOR were determined by western blots in BMDMs of *Pgam5* cKO and *Pgam5*^fl/fl^ mice. Relative protein levels with statistical analyses were listed in Fig. S7a (*n* = 3). **b** qPCR analysis of iNOS, IL1α, IL1β and IL-6 mRNA levels in *Pgam5* cKO and *Pgam5*^fl/fl^ macrophages after M1 induction with or without treatment of MHY1485 (10 μmol/L). **c** Western blot analysis of iNOS protein level of *Pgam5* cKO and *Pgam5*^fl/fl^ macrophages after M1 induction with or without treatment of MHY1485 (10 μM). Relative protein levels with statistical analyses were listed in Fig. S7b (*n* = 3). **d** Western blot analysis of p-mTOR, mTOR, and iNOS protein levels in BMDMs with increasing concentrations of MHY1485 stimulation. Relative protein levels with statistical analyses were listed in Fig. S7c (*n* = 3). **e** qPCR analysis of iNOS, IL1α, IL1β and IL-6 mRNA levels in *Pgam5* cKO BMDMs treated with increasing concentrations of MHY1485 stimulation. **f** Western blot analysis of PPARγ and p-STAT6 protein levels in *Pgam5*^fl/fl^ and *Pgam5* cKO BMDMs after 24 h of stimulation with IL4. Relative protein levels with statistical analyses were listed in Fig. S7d (*n* = 3). **g** Western blot analysis of PPARγ and CD206 protein levels in *Pgam5* cKO BMDMs with increasing concentrations of T0070907 stimulation. Relative protein levels with statistical analyses were listed in Fig. S7e (*n* = 3). **h** qPCR analysis of Arg1, Ym1, and CD206 mRNA levels in *Pgam5* cKO macrophages after M2 induction with or without treatment of T0070907 (5 μmol/L). **i** Western blot analysis of CD206 protein level in *Pgam5* cKO and *Pgam5*^fl/fl^ macrophages after M2 induction with or without treatment of T0070907 (5 μmol/L). Relative protein levels with statistical analyses were listed in Fig. S7f (*n* = 3). **j** Western blot analysis of CD206 and PPARγ protein levels in *Pgam5* cKO and *Pgam5*^fl/fl^ macrophages after M2 induction with or without treatment of AS1517499 (10 μmol/L). Relative protein levels with statistical analyses were listed in Fig. S7g (*n* = 3). Data are shown as the mean±s.d. *P < 0.05, **P < 0.01, ***P < 0.001 between the indicated groups. *P* values were determined using Student’s *t* tests

In previous studies, a series of signaling pathways were activated by induction of M1 polarization, such as the AKT-mTOR and MAPK signaling pathways.^12^ We further examined whether PGAM5 promotes M1 polarization through these specific signals. Treated with LPS and IFNγ, BMDMs from *Pgam5* cKO mice significantly showed lower protein levels of p-p38 and p-ERK than *Pgam5*^fl/fl^ macrophages and nearly identical expression of total p38 and total ERK, indicating that PGAM5 activates the p38 and ERK signaling pathways to enhance M1 polarization since MAPK signaling has been proven to increase M1 polarization in previous studies^15^ (Fig. 5a). However, we did not find different expression pattern of p-JNK in *Pgam5* cKO and *Pgam5*^fl/fl^ BMDMs (Fig. 5a). p-AKT and p-mTOR significantly decreased in *Pgam5* cKO macrophages compared with *Pgam5*^fl/fl^ macrophages stimulated by LPS and IFNγ (Fig. 5a). To explore the possible role of AKT and mTOR in mediating the intensive role of PGAM5 in macrophage inflammation, we employed the specific mTOR activator MHY1485^16^ to activate the mTOR pathway. Activating mTOR rescued the decreased levels of iNOS, IL1α, IL1β and IL-6 in PGAM5-deficient macrophages after LPS and IFNγ stimulation, as examined by qPCR (Fig. 5b), indicating that mTOR activity is involved in the regulation of M1 macrophage polarization by PGAM5. The protein level of iNOS was also enhanced in the presence of MHY1485 in *Pgam5* cKO macrophages (Fig. 5c). The activation of mTOR, also presented by phosphorylation of mTOR, increased in BMDMs when treated with doses of 0, 1, 5, and 10 μmol/L of MHY1485 (Fig. 5d), and the mRNA levels of iNOS, IL1α, IL1β, and IL6 increased in *Pgam5* cKO macrophages in a dose-dependent manner (Fig. 5e), indicating that PGAM5 promoted the M1 phenotype by activating the mTOR signaling pathway.

To explore the mechanisms by which PGAM5 modulates M2 polarization, we focused on the STAT6-PPARγ signaling pathway, which regulates M2 polarization.^17^ After stimulation with IL4 for 24 h, the protein levels of PPARγ and p-STAT6 enhanced in *Pgam5*^-^cKO BMDMs compared to *Pgam5*^fl/fl^ BMDMs (Fig. 5f), indicating that the STAT6-PPARγ signaling pathway might be involved in PGAM5-mediated M2 polarization. Next, inhibition of PPARγ by a specific inhibitor, T0070907,^18^ markedly reduced the protein level of CD206 in a dose-dependent manner (Fig. 5g). Furthermore, T0070907 decreased the M2 polarization response in *Pgam5* cKO macrophages, as indicated by significantly decreased mRNA levels of Arg1, chitinase-like 3 (Ym1), and CD206 (Fig. 5h). Besides, the protein level of CD206 was also markedly reduced by treatment with T0070907 in *Pgam5* cKO macrophages stimulated by IL4 (Fig. 5i), suggesting that M2 polarization was regulated by PGAM5 through the STAT6-PPARγ signaling pathway. To identify the role of STAT6 in the PGAM5 modulation of M2 polarization, specific inhibition of STAT6 by AS1517499^19^ was added to *Pgam5* cKO and *Pgam5*^fl/fl^ macrophages at a dose of 10 μmol/L. As a result, the protein expression of PPARγ and CD206 significantly reduced, indicating that STAT6 functions as a regulator of PPARγ (Fig. 5j). In conclusion, PGAM5 regulates M2 polarization via the STAT6-PPARγ signaling pathway.

### PGAM5 regulated macrophage polarization by targeting the β-catenin pathway via dephosphorylation of DVL2

Although we have identified the related signaling pathways that regulate PGAM5-mediated macrophage polarization, the direct target of PGAM5 in regulating macrophage polarization has not been verified. PGAM5 was reported to inhibit the Wnt/β-catenin signaling pathway on the mitochondrial membrane in human cells and Xenopus embryogenesis.^20^ Moreover, the β-catenin signaling pathway is closely correlated with macrophage activation and polarization.^21–23^ Nevertheless, whether PGAM5 modulates β-catenin in synovial macrophage polarization and the related targets have not been reported.

**Fig. 6:**
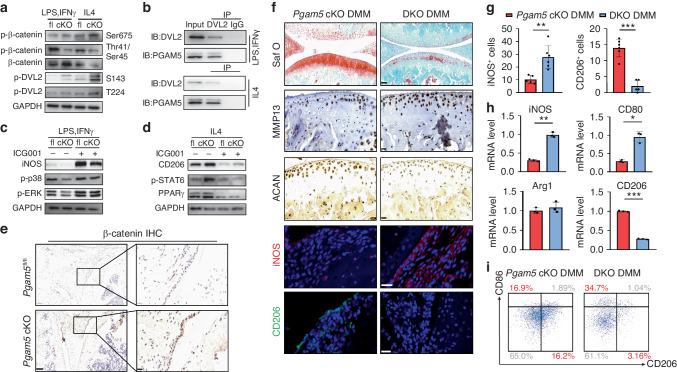
PGAM5 modulated macrophage polarization by inhibiting the β-catenin signaling pathway via dephosphorylation of DVL2. **a** Western blot analysis of p-β-catenin on Ser675 and Thr41/Ser45, β-catenin and p-DVL2 on S143 and T224 protein levels in *Pgam5*^fl/fl^ and *Pgam5* cKO BMDMs after stimulation with LPS plus IFNγ or IL4. Relative protein levels with statistical analyses were listed in Fig. S8a (*n* = 3). **b** Coimmunoprecipitation of DVL2 and PGAM5 in M1 and M2 macrophages. **c** Western blot analysis of iNOS, p-p38, and p-ERK protein levels in *Pgam5*^fl/fl^ and *Pgam5* cKO macrophages stimulated with ICG-001 (10 μmol/L). Relative protein levels with statistical analyses were listed in Fig. S8b (*n* = 3). **d** Western blot analysis of CD206, p-STAT6, and PPARγ protein levels in *Pgam5*^fl/fl^ and *Pgam5* cKO macrophages stimulated with ICG-001 (10 μmol/L). Relative protein levels with statistical analyses were listed in Fig. S8c (*n* = 3). **e** Immunohistochemistry of β-catenin in the OA synovium of *Pgam5*^fl/fl^ and *Pgam5* cKO mice after DMM surgery. **f** Representative images of safranin O staining (scale bars, 50 µm), immunohistochemistry of ACAN, MMP13, immunofluorescence of iNOS (red) and CD206 (green) (scale bars, 20 µm) of DKO and *Pgam5* cKO mice 28 days after DMM surgery (*n* = 6). **g** Quantification of iNOS (red) and CD206 (green) immunofluorescence in the synovium of the knee joint of DKO and *Pgam5* cKO mice 28 days after DMM surgery. **h** qPCR analysis of iNOS, CD80, Arg1, and CD206 mRNA levels in peritoneal macrophages of DKO and *Pgam5* cKO mice 28 days after DMM surgery. **i** Flow cytometry of peritoneal macrophages of DKO and *Pgam5* cKO mice 28 days after DMM surgery. Data are shown as the mean±s.d. *P < 0.05, **P < 0.01, ***P < 0.001 between the indicated groups. *P* values were determined using Student’s *t* tests

Thus, we first examined the potential regulation of PGAM5 on β-catenin in macrophage polarization. *Pgam5* cKO and *Pgam5*^fl/fl^ BMDMs were isolated and stimulated to induce M1 or M2 polarization. After 24 h of stimulation, *Pgam5* cKO BMDMs showed increased phosphorylation of β-catenin at Ser675 and decreased phosphorylation at Thr41/Ser45 in the M1- and M2-polarized states, and increased protein levels of β-catenin were detected in the M1-polarized state compared to *Pgam5*^fl/fl^ BMDMs, indicating that PGAM5 triggered inhibition of β-catenin in both M1 and M2 polarization (Fig. 6a). To better confirm the target of PGAM5 on β-catenin in macrophage polarization, we focused on whether PGAM5 could dephosphorylate Dishevelled Segment Polarity Protein 2 (DVL2), which is a inhibitory regulator in the upstream of β-catenin signaling pathway. Interestingly, the phosphorylation sites of DVL2, S143 and T224, were both increased in *Pgam5* cKO macrophages when induced to M1 and M2-polarized states compared to *Pgam5*^fl/fl^ BMDMs (Fig. 6a), indicating potential interaction of PGAM5 and DVL2. Thus, we detected whether PGAM5 could directly bind to DVL2 and found that PGAM5 could be coimmunoprecipitated with DVL2 in both M1 and M2 macrophages (Fig. 6b), indicating PGAM5 directly dephosphorylated DVL2 via binding to it. We then focused on whether β-catenin was involved in PGAM5-modulated macrophage polarization. Specific inhibition of β-catenin by ICG-001^24^ increased the protein levels of p-p38, p-ERK and iNOS. ICG-001 also reduced the expression of p-STAT6, PPARγ, and CD206, indicating that β-catenin functions as a regulator of macrophage polarization (Fig. 6c, d). In addition, as predicted, the expression of β-catenin increased in the OA synovium of *Pgam5* cKO mice compared to *Pgam5*^fl/fl^ mice (Fig. 6e). Thus, these results indicated that PGAM5 modulated macrophage polarization by inhibiting the β-catenin pathway via directly targeting DVL2.

To verify the role of β-catenin in PGAM5-mediated macrophage polarization in synovium, we generated mice in which both PGAM5 and β-catenin were ablated in macrophages, herein referred to as DKO mice, by crossing *β-catenin*^fl/fl^ mice with *Pgam5* cKO mice. Knee joints of DKO and *Pgam5* cKO male mice were collected 28 days after DMM surgery for further investigations. DKO mice significantly exacerbated OA symptoms compared to *Pgam5* cKO mice, as determined by safranin O staining (Fig. 6f). OARSI scores of safranin O staining were listed in the supplementary materials (Fig. S3). Expression of ACAN decreased while expression of MMP13 increased in the knee joint of DKO mice compared to *Pgam5* cKO mice, indicating that the relieved OA symptom by PGAM5 deficiency in macrophages was partly mediated by the enhanced activity of β-catenin (Fig. 6f). In addition, iNOS-positive cells were significantly increased and CD206^+^ cells were significantly decreased in the synovium of DKO mice compared to *Pgam5* cKO mice (Fig. 6f, g), suggesting that PGAM5 regulates synovial macrophage polarization by inhibiting the β-catenin signaling pathway, further aggravating the progression of OA. Peritoneal macrophages in DKO mice showed increased mRNA levels of iNOS and CD80 and decreased mRNA levels of CD206 (Fig. 6h). In addition, flow cytometry of peritoneal macrophages also indicated that DKO mice showed more proinflammatory phenotypes than *Pgam5* cKO mice (Fig. 6i). In conclusion, PGAM5 regulates macrophage polarization by inhibiting the β-catenin signaling pathway, and inhibition of β-catenin extensively reversed the alleviation of OA symptoms in *Pgam5* cKO mice.

### Targeted knockdown of PGAM5 in synovial macrophages by MFP9-2/siPGAM5 relieved OA symptoms

**Fig. 7:**
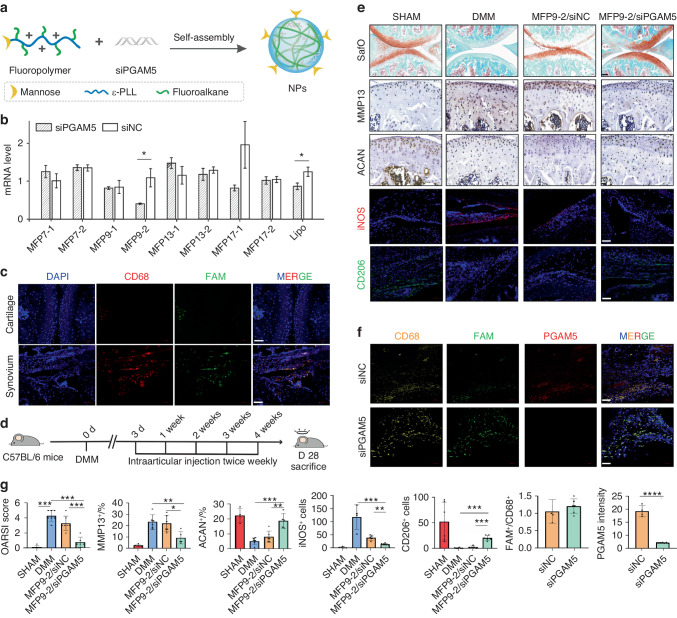
Intraarticular injection of macrophage-targeted siPGAM5 delivery system to relieve OA symptoms. **a** Preparation of macrophage-targeted nanoparticles (NPs). **b** qPCR analysis of PGAM5 mRNA level in Raw264.7 cells after transfected with the polymer/siRNA complexes for 24 h. The concentrations of siRNA and the polymers were 8 µg/mL and 16 µg/mL, respectively. **c** Communofluorescence of CD68 (red), FAM (green), and DAPI (blue) in the cartilage and synovium of mice 28 days after DMM surgery with injection of MFP9-2/siPGAM5 (scale bars, 50 µm). **d** Schematic diagram of intervention of OA by articular injection of MFP9-2/siPGAM5 twice weekly after DMM surgery. **e** Representative images of safranin O staining (scale bars, 100 µm), immunohistochemistry of MMP13, and ACAN (scale bars, 20 µm), and immunofluorescence of iNOS (red)and CD206 (green) (scale bars, 50 µm) in knee joints 28 days after DMM surgery (*n* = 6). Sham group, DMM group with no injection and DMM group with injection of MFP9-2/siNC were established as controls. **f** Coimmunofluorescence of CD68 (yellow), FAM (green), and PGAM5 (red) in the synovium of mice 28 days after DMM surgery with injection of MFP9-2/siNC or MFP9-2/siPGAM5 (scale bars, 50 µm). **g** OARSI scores, the percentage of MMP13 positive cells, ACAN positive cells and the amounts of iNOS positive cells and CD206 positive cells were quantified in each group. Ratio of FAM positive cells and CD68 positive cells, and the mean IF intensity of PGAM5 were quantified. Data are shown as the mean±s.d. *P < 0.05, **P < 0.01, ***P < 0.001, ****P < 0.000 1 between the indicated groups. *P* values were determined using Student’s *t* tests

Based on the mechanisms of PGAM5 in regulating macrophage polarization, we aimed to establish targeted deletion of PGAM5 in synovial macrophages to treat OA in early stage. RNA interference (RNAi) is a powerful technique to treat various diseases via specific gene silence.^25^ However, targeted delivery of siRNA into synovial macrophages is challenging owing to the complicated synovial fluid composition and extracellular matrix in the joint microenvironment.^26^ Here, we developed a series of mannose modified fluoropolymers for macrophage-targeted siRNA delivery to treat OA via inhibition of PGAM5. ε-PLL was conjugated with fluoroalkanes (F7-F17, Fig. 7a) via amine-epoxide reactions, and different feeding ratios were chosen to get PLL modified with average numbers of about 10 and 15 fluoroalkanes, respectively. The obtained fluoropolymers were further grafted with mannose via an amine-isocyanate reaction, and an average number of 1 mannose was conjugated on each polymer calculated by ^1^H NMR. Take F7 for example, the mannose grafted polymer conjugated with 10 F7 ligands and 15 F7 ligands were termed MFP7-1 and MFP7-2, respectively. The siRNA delivery efficacy of the synthesized nanoparticles (NPs) was first screened on Raw264.7 cells. MFP9-2/siPGAM5 complexes exhibited the highest gene knockdown efficiency, which was higher than that of the Lipofectamine 2000 (Lipo)/siPGAM5 complexes (Fig. 7b). The MFP9-2 transported the FAM-labeled siRNA into the CD68^+^ synovial macrophages rather than chondrocytes efficiently after intraarticular injection (Fig. 7c), which is beneficial for achieving macrophage-targeted RNAi. To validate the therapeutic efficacy of the MFP9-2 based siRNA delivery system, MFP9-2/siPGAM5 complex was injected into the joint of WT mice twice weekly in early stage of OA established by DMM surgery and the joints were collected 28 days after DMM surgery. Sham group, DMM group with no injection and DMM group with injection of MFP9-2 combined with siNC (MFP9-2/siNC) were established as controls (Fig. 7d). Intraarticular injection of MFP9-2/siPGAM5 greatly relieved the OA symptoms compared to DMM group and DMM with injection of MFP9-2/siNC group, indicated by increased safranin O staining area, decreased expression of MMP13 and enhanced level of ACAN in cartilage (Fig. 7d). Besides, the amount of iNOS positive cells in synovium treated with MFP9-2/siPGAM5 greatly decreased, while CD206 positive cells increased compared to DMM group and DMM with injection of MFP9-2/siNC group (Fig. 7e, g), indicating the availability of macrophage modulation in OA synovium by MFP9-2/siPGAM5. To further confirm the modulatory function of MFP9-2/siPGAM5, we detected whether the relief of OA symptoms was the result of PGAM5 inhibition in synovial macrophage by MFP9-2/siPGAM5. We found that injection of MFP9-2/siPGAM5 greatly decreased the level of PGAM5 in CD68 positive macrophages in synovium compared to injection of MFP9-2/siNC with FAM (Fig. 7f, g), indicating successful targeting and inhibiting of PGAM5 in synovial macrophages, which could further achieve better OA outcomes.

To conclude, PGAM5 serves as a novel factor of regulating macrophage polarization in osteoarthritis via dephosphorylating DVL2, resulting in increased activity of GSK3β and degradation of β-catenin,^27^ which disables the translocation of β-catenin into nucleus to bind to promotors for downstream signaling pathways, further contributes to increased M1 and decreased M2 phenotypes via specific signals. To better treat OA via early intervention of macrophage PGAM5, specifically inhibition of PGAM5 in macrophages was achieved by intraarticular injection of MFP9-2/siPGAM5, which could significantly target synovial macrophages and reduce the expression of PGAM5 in macrophages, resulting in the relief of OA symptoms. Together, we have clarified the modulatory role of PGAM5 in OA macrophage and designed a functional macrophage-targeted therapy, which might contribute to early and precise immunological interventions in OA in clinic.

## Discussion

Early intervention of OA has been focused to gain better clinical outcomes, which requires for identification of molecular biomarkers and validation of molecular targets for novel targeted therapies in the early-stage OA.^28^ In the OA pathophysiology, synovial macrophages exhibit distinct functions.^29^ Macrophages showed distinct phenotypes of M1 and M2 macrophages. M1 macrophages are also termed proinflammatory macrophages, which are activated by LPS produced by microbes or IFNγ and characterized by the production of inflammatory cytokines such as IL-1, IL-6, IL-12, TNF-α and iNOS, which promote the Th1 response.^14,17,30^ In contrast, M2 macrophages showed anti-inflammatory functions by promoting the Th2 response and enhanced tissue remodeling. M2 macrophages can be alternatively activated by IL-4, IL-13, glucocorticoids, IL-10, and immunoglobulin complexes/Toll-like receptor ligands and are characterized by enhanced expression of multiple cell surface markers, including mannose receptor Mrc1 (also known as CD206), CD9, and CD36. They also produce various cytokines, like arginase (Arg), IL-10, IL-1 receptor antagonist (IL-1ra), and the type II IL-1 decoy receptor.^14,30,31^ Since investigations have shown that synovial macrophage polarization is closely associated with OA,^5,32,33^ precise therapies for macrophage to decrease synovitis and attenuate OA progression need to be clearly proposed.

In this study, we found that PGAM5 significantly increased in macrophages in OA synovium compared to controls based on histology of human samples and single-cell RNA sequencing results of mice models. Accordingly, we constructed DMM model in transgenic mice with macrophage-specific deletion of PGAM5 to identify the modulatory role of PGAM5 in synovial macrophages. We observed alleviated OA symptoms in *Pgam5* cKO mice compared to controls due to repolarization of M1 macrophages into M2 macrophages in synovium. Additionally, *Pgam5* cKO BMDMs tilted polarization toward M2 macrophages, which promoted anabolic metabolism of chondrocytes in vitro. To clarify the underlying mechanisms of macrophage polarization regulated by PGAM5 in OA, we examined macrophage-related signalings in *Pgam5* cKO BMDMs. In previous studies, Akt-mTOR signaling pathway is significantly activated in the process of M1 polarization by stimulation of LPS and IFNγ, in which Akt activation could lead to the inactivation of tuberous sclerosis complex (TSC) 1/2, a critical factor for M2 polarization and attenuation of M1 responses,^31^ further activating mTORC1 and increasing the M1 phenotype.^34^ Thus, we examined Akt-mTOR signaling in *Pgam5* cKO BMDMs during M1 induction and found that the decreased M1 response of *Pgam5* cKO macrophages was partly due to reduced activation of the Akt-mTOR signaling pathway. However, Jin Fan et al. also revealed that the activation of Akt pathway could enhance M2 macrophage polarization,^35^ indicating the different mechanisms of Akt signaling in modulating macrophage polarization. To clarify the intracellular pathways involved in PGAM5-mediated M1 polarization, we used MHY1485, a specific mTOR agonist,^16^ to activate mTOR signaling in *Pgam5* cKO macrophages. As a result, MHY1485 significantly reversed the decreased M1 phenotype of *Pgam5* cKO macrophages in a dose-dependent manner, indicating PGAM5 induced M1 polarization via activating Akt-mTOR signaling. Furthermore, we detected whether PGAM5 also activated MAPK-related signaling to modulate M1 polarization, since p38/STAT3, ERK/MEK, and JNK signals are proved activated by M1-correlated stimuli such as LPS, serving as signal transducers to enhance the M1 response.^31,36–39^ We then proved that PGAM5 deletion in macrophages could inactivate p38 and ERK signaling when treated with LPS and IFNγ, suggesting that p38/ERK MAPK signaling pathways participated in PGAM5-induced M1 polarization. Similarly, we revealed that PGAM5 inhibited M2 polarization by STAT6-PPARγ by usage of STAT6 inhibitor (AS1517499) and PPARγ inhibitor (T0070907). These results verified that PGAM5 is a critical regulator of macrophage polarization. However, we were not able to obtain Pgam5 OE(overexpression) mice and further focused on the knockout of PGAM5 in macrophages as a promising therapeutics in clinic.

In previous studies, PGAM5 was shown to play a complex role of modulating β-catenin signaling pathway. DVL2 serves as a substrate of PGAM5 and could be dephosphorylated by PGAM5 directly on the mitochondrial membrane due to its phosphatase activity, which activates GSK3β, an antagonist of the Wnt-β-catenin signaling pathway, by phosphorylating β-catenin on Thr41 and inhibiting its activity,^20,40,41^ indicating that PGAM5 functions as an inhibitor of the β-catenin signaling pathway. Nevertheless, some studies also proved that PGAM5 could directly bind to β-catenin and activate the Wnt-β-catenin signaling pathway.^42,43^ To further verify the association between PGAM5 and β-catenin signaling during synovial macrophage polarization, we further examined the expression patterns of potential targets of PGAM5. Our observations showed that the levels of β-catenin and p-DVL2 were enhanced in *Pgam5* cKO macrophages during M1 and M2 induction, suggesting that PGAM5 might directly dephosphorylate DVL2 to inhibit the β-catenin pathway in macrophages. Next, co-IP of PGAM5 and DVL2 indicated that PGAM5 directly binds to DVL2 and leads to inhibition of the β-catenin pathway. Accordingly, inhibition of the β-catenin pathway by ICG-001, a specific inhibitor of the β-catenin pathway,^24^ significantly enhanced the M1 phenotype by activating the p38/ERK signaling pathway and reduced the M2 response by inhibiting the STAT6-PPARγ pathway in vitro. Furthermore, *Pgam5*/catenin DKO mice showed increased OA symptoms compared to *Pgam5* cKO mice, indicating that PGAM5 promoted M1 polarization and inhibited M2 polarization of synovial macrophages by downregulating β-catenin both in vitro and in vivo.

Motivated by these findings, we aimed to deliver siRNA targeting PGAM5 in synovial macrophages to treat OA. Few studies have achieved macrophage-targeted delivery of siRNA in OA synovium, since siRNA is a large hydrophilic molecule which is difficult to penetrate across the semipermeable cell membrane, resulting in the low efficacy of intracellular delivery.^44^ Besides, the negatively charged siRNA could be easily degraded in the cytoplasm leading to short residence of siRNA.^44^ Accordingly, we constructed mannose modified fluoropolymers combined with siPGAM5 and treated mice via intraarticular injection. The fluoropolymers modified by fluoroalkanes have excellent advances in gene delivery,^45^ since the strong hydrophobicity of fluoropolymers ensures efficient cell membrane penetration and endosomal escape of siRNA, and cationic polymers by fluorination protect nucleic acids from enzyme degradation. As a result, we detected stable residency of FAM-modified siPGAM5 in synovial macrophages up to a week. Furthermore, fluoropolymers could be easily modified by mannose for targeting synovial macrophages, owing to the widely expressed mannose receptors on the surface of macrophages.^46^ Amazingly, we found that nearly a hundred percent of synovial macrophages were targeted and PGAM5 in macrophages was inhibited via the intraarticular injection of siPGAM5 NPs, leading to repolarization of M1 macrophages into M2 macrophages which greatly alleviated OA. What’s more, the cytotoxicity of fluoropolymers is less than other cationic polymers due to less nitrogen to phosphorus ratio,^44^ which is a promising therapeutic for targeting synovial macrophages in clinical intervention.

In summary, we demonstrated that PGAM5 directly interacted with DVL2 to modulate the β-catenin pathway, further amplifying the M1 response via the Akt-mTOR, p38, and ERK signaling and inhibiting M2 polarization mediated by downregulating the STAT6-PPARγ pathway. Accordingly, targeted knockdown of PGAM5 in macrophages by macrophage-targeted siPGAM5 NPs greatly relieved OA symptoms in mice. This immune-targeted approach promoted anabolic and inhibited catabolic metabolism of chondrocytes, which offered a new avenue for OA intervention in addition to traditional pharmacological strategies and regenerative therapies.^47^ Furthermore, the critical function of PGAM5 in OA might provide novel clinical targets and the related macrophage-targeted therapy will offer new therapeutic strategies of precise immunomodulation in OA for better outcomes.

## Materials and methods

### Animals

Myeloid cell-specific PGAM5 conditional knockout mice (*Pgam5* cKO mice) were obtained by crossing PGAM5loxp/loxp mice with mice expressing Cre recombinase under the control of the Lysozyme promoter (Lyz2). Myeloid cell-specific PGAM5 and β-catenin conditional knockout mice (DKO mice) were obtained by crossing β-cateninloxp/loxp mice with *Pgam5* cKO mice. Lyz2Cre-negative, PGAM5loxp/loxp and β-cateninloxp/loxp littermates served as the controls. Eight-week-old mice (male) were usually used for the in vitro experiments. PGAM5loxp/loxp and Lyz2Cre mice were generous gifts from Prof. Wei Lu.^8^ β-cateninloxp/loxp mice were kindly provided by Prof. Xuefeng Wu.^48^ All mice were maintained in a specific pathogen-free facility. All experimental manipulations were undertaken in accordance with the Institutional Guidelines for the Care and Use of Laboratory Animals, Institute of Zoology (Shanghai, China).

### Human synovium

Normal human synovium was obtained from bone fracture patients with no history of arthritic diseases (*n* = 3). Human OA synovium was obtained from patients undergoing total knee replacement surgery (*n* = 5). Human samples were obtained from Shanghai Xinhua Hospital and Shanghai Dongfang Hospital. All patients gave informed consent to use their clinical information for scientific research. The study was approved by the Ethics Committee of the Shanghai Xinhua Hospital and Shanghai Dongfang Hospital.

### Reagents

Anti-mCD86-PE/Cy5 and anti-mCD206-FITC were purchased from Cell Signaling Technology. Bacterial lipopolysaccharide (LPS; E. coli 055: B5) was purchased from Sigma‒Aldrich. Recombinant mouse IL-4 and IFNγ were purchased from PeproTech (Rocky Hill, NJ). An agonist of mTOR (MHY1485), inhibitor of PPARγ (T0070907) and inhibitor of STAT6 (AS1517499) were purchased from Selleck. Primary antibodies against ERK, p-ERK1/2 (Thr202/Tyr204), p-JNK (Thr183/Tyr185), P38, p-p38 (Thr180/Tyr182), Akt, p-Akt (Thr308), p-Akt (Ser473), STAT6, p-STAT6 (Tyr641), p-β-catenin (Ser675, Thr41/Ser45) and β-tubulin were purchased from Cell Signaling Technology. Primary antibodies against PGAM5, PPARγ, MMP13, β-catenin, DVL2 and p-DVL2 (S143, T224) were purchased from ABclonal. ACAN antibody was purchased from ABclonal (A11691; Wuhan, China). All of these antibodies were diluted at 1:1 000 in 5% bovine serum albumin (BSA). ELISA was performed using the Mouse IL-12p70 ELISA Kit (EK212/3-96, Lianke), IL-6 Mouse Uncoated ELISA Kit (88-7064-88, Thermo Fisher), and Mouse IL-1 beta Uncoated ELISA (88-7013-88, Thermo Fisher).

### Cell preparation

Bone marrow cells were cultured with Dulbecco’s modified Eagle medium (DMEM) containing 10% (v/v) FBS and 10 ng/mL mouse M-CSF or 10 ng/mL mouse GM-CSF for 12 days to obtain BMDMs. The nonadherent cells were removed by washing with PBS. The inflammatory response of macrophages was induced by LPS (100 ng/mL) and IFNγ (50 ng/mL) for 24 h. M2 macrophages were induced by IL-4 (1 000 U/mL) treatment for 24 h.

### Quantitative PCR analysis

Total RNA was extracted by transfer to TRIzol reagent (Invitrogen, Waltham, MA) from 6-well plates and then homogenized at high speed on ice. DNase I (Sigma‒Aldrich, St. Louis, MO) was added to the extracted mRNA to remove genomic DNA. The quantification of mRNA was performed and calculated using a Nanodrop 2000 (Thermo Fisher Scientific). mRNA was reversely transcribed into complementary DNA (cDNA) in each experimental and control group using the PrimeScript RT Master Mix Kit (Takara Bio Inc., Dalian, China). Then, cDNA was tested by PCR via the SYBR Premix Ex Taq Kit (RR420a; Takara, Tokyo, Japan). To normalize the mRNA expression, the level of the housekeeping gene GAPDH served as a control. Quantitative PCR (qPCR) primers for the genes and forwards (F) and reverse (R) primer sequences were listed in Table 1.

**Table 1:** Primer sequences for qPCR

Gene	Primer sequences
hPGAM5-F	TCGTCCATTCGTCTATGACGC
hPGAM5-R	GGCTTCCAATGAGACACGG
hGAPDH-F	GGAGCGAGATCCCTCCAAAAT
hGAPDH-R	GGCTGTTGTCATACTTCTCATGG
mCOL2A1-F	ACGAGGCAGACAGTACCTTG
mCOL2A1-R	CAGCCCTGGTTGGGATCAAT
mMMP13-F	TTGGCTTAGAGGTGACTGGC
mMMP13-R	CCACATCAGGCACTCCACAT
mSOX9-F	TCAGCAAGACTCTGGGCAAG
mSOX9-R	TCCGTTCTTCACCGACTTCC
mMMP3-F	CCACTCCCTGGGACTCTAC
mMMP3-R	TGAGAGAGATGGAAACGGGAC
mPGAM5-F	CCCTGCAAGAAGACTGTGGT
mPGAM5-R	GTCAGCGGGGGCTAAATCTT
mGAPDH-F	TGACCTCAACTACATGGTCTACA
mGAPDH-R	CTTCCCATTCTCGGCCTTG
mCD80-F	TCAGTTGATGCAGGATACACCA
mCD80-R	AAAGACGAATCAGCAGCACAA
mIL1β-F	GCAACTGTTCCTGAACTCAACT
mIL1β-R	ATCTTTTGGGGTCCGTCAACT
mTNF-F	CCCTCACACTCAGATCATCTTCT
mTNF-R	GCTACGACGTGGGCTACAG
mIL10-F	CTTACTGACTGGCATGAGGATCA
mIL10-R	GCAGCTCTAGGAGCATGTGG
mIL4-F	GGTCTCAACCCCCAGCTAGT
mIL4-R	GCCGATGATCTCTCTCAAGTGAT
mIL6-F	CTGCAAGAGACTTCCATCCAG
mIL6-R	AGTGGTATAGACAGGTCTGTTGG
mIL1A-F	AGTATCAGCAACGTCAAGCAA
mIL1A-R	TCCAGATCATGGGTTATGGACTG
mIl1b-F	GAAATGCCACCTTTTGACAGTG
mIl1b-R	TGGATGCTCTCATCAGGACAG
mIL12-F	TGGTTTGCCATCGTTTTGCTG
mIL12-R	ACAGGTGAGGTTCACTGTTTCT
mPPARγ-F	GGAAGACCACTCGCATTCCTT
mPPARγ-R	GTAATCAGCAACCATTGGGTCA
mArg1-F	TGTCCCTAATGACAGCTCCTT
mArg1-R	GCATCCACCCAAATGACACAT
mFizz1-F1	CTGCCCTGCTGGGATGACT
mFizz1-R1	CATCATATCAAAGCTGGGTTCTCC
mYm1-F1	CAAGTTGAAGGCTCAGTGGCTC
mYm1-R1	CAAATCATTGTGTAAAGCTCCTCTC

### Immunohistochemistry and pathological staining

ACAN, MMP13, β-catenin, iNOS and CD206 in pathological sections were examined by immunohistochemistry. Paraffin sections of joints were dewaxed, rehydrated, pretreated with pepsin at 37 °C for 30 min, and then incubated with 3% H_2_O_2_ in methanol solution. After rinsing with PBS, the slices were blocked with BSA at room temperature for 1 hour and then incubated overnight with primary antibodies at 4 °C. Then, the slices were incubated with the secondary antibody provided in the HRP polymer anti-rabbit IHC kit (Kit 5005; MaxVision, Shenzhen, China) for 15 min and stained with substrate from the DAB Plus kit (DAB-2031) for 10 min. Histological images of the knee joint were obtained after staining with safranin O and fast green. We performed immunofluorescence using a multicolored immunofluorescence kit (abs50012, absin) and observed the cells using a microscope from Zeiss.

### Western blot assay and coimmunoprecipitation

Macrophages were cultured in DMEM with 10% FCS in 6-well plates. Cells were treated with IL-4 (100 nmol/L) or LPS (100 ng/mL) for the indicated times. After stimulation, cells were washed once in cold PBS and lysed in RIPA buffer (50 mmol/L Tris–HCl pH 7.4, 1% NP-40, 0.25% Na-deoxycholate, 150 mmol/L NaCl, 1 mmol/L EDTA pH 7.4) with protease and phosphatase inhibitor cocktails (Sigma) for 10 min on a rocker at 4 °C. Protein concentration was determined using a BCA assay. Protein samples were analysed by SDS polyacrylamide gel electrophoresis (SDS–PAGE) and transferred onto PVDF membranes (Millipore, CA)60. Each polyvinylidene fluoride membrane was blocked with TBST (100 mmol/L Tris–HCl pH 7.5, 150 mmol/L NaCl, 0.05% Tween 20) with 5% BSA for 1 h and then incubated with primary antibodies overnight on a shaker at 4 °C. The appropriate HRP-coupled secondary antibody was then added and detected through chemiluminescence (Millipore). GAPDH and β-tubulin were used as protein loading controls. Coimmunoprecipitation was performed with indicated antibodies and G/A beads following information of co-IP kit (absin, abs955). Quantitative analyses with a *p-*value between indicated groups in the WB were calculated via ImageJ.

### Flow cytometry

Primary BMDMs were induced under specific conditions and labeled with the following antibodies for identification of M1 or M2 macrophages: PE/Cyanine7 anti-mouse F4/80 Antibody (Biolegend, 123113) PE/Cyanine5 anti-mouse CD86 Antibody (Biolegend, 105015), FITC anti-mouse CD206 (MMR) Antibody (Biolegend, 141703). Related isotypes for control are PE/Cyanine7 Rat IgG2a, κ Isotype Ctrl Antibody (Biolegend, 400521), PE/Cyanine5 Rat IgG2a, κ Isotype Ctrl Antibody (Biolegend, 400509), FITC Rat IgG2a, κ Isotype Ctrl Antibody (Biolegend, 400505). Cells were suspended and incubated with antibodies for 30 min, and analyzed via Guava Easycyte 12HT (Luminex).

### siRNAs

siRNAs targeting mouse PGAM5 with FAM labeled were purchased from Sangon Biotech. Scrambled siRNA with FAM labeled was used as a control. Raw264.7 cells were transfected with siRNA using Lipofectamine 2000 (Invitrogen, Thermo Fisher Scientific, Waltham, MA, USA) in serum-free conditional medium for examination. Sequence of siRNA targeting PGAM5 is: sense (5’-3’) : (FAM)CUGGAGAAGACGAGUUGACAUTT, antisense (5’-3’) : AUGUCAACUCGUCUUCUCCAGTT. Experiments were repeated for at least three times independently.

### Synthesis of mannose modified fluoropolymers

ε-PLL (Mw: 4 224 Da) was mixed with epoxides bearing fluoroalkanes at different molar ratios in anhydrous methanol, and stirred at 60 °C for 48 h. The crude products were dialyzed against methanol and distilled water, then lyophilized to obtain the fluoropolymers. The average number of fluoroalkanes modified on each polymer was tested by a well-established ninhydrin assay^49^ (Fig. S9). The obtained fluoropolymers were further reacted with d-mannopyranosylphenyl isothiocyanate in dimethylsulfoxide at a molar ratio of 1:1 at room temperature for 24 h, then lyophilized to obtain the mannose modified fluoropolymers. The average number of mannoses modified on each polymer was characterized and calculated by ^1^H-NMR (Bruker, 500 MHz).

### Statistical analysis

All data are presented as the mean ± s.d. Two-way ANOVA was used for comparisons among multiple groups with SPSS 16.0 software. Student’s unpaired *t* test for comparison of means was used to compare two groups. Log-rank tests were used for mouse survival assays. A *P* value less than 0.05 was considered to be statistically significant.

### Supplementary information

Supplementary materials of "Targeted knockdown of PGAM5 in synovial macrophages efficiently alleviates osteoarthritis"

## Data Availability

All data needed to evaluate the conclusions in the paper are present in the paper and/or the Supplementary Materials. Data will be made available upon reasonable request.
