# Supplementary material for: Targeted knockdown of PGAM5 in synovial macrophages efficiently alleviates osteoarthritis
Source: Bone Res. 2024 Mar 4;12:15. doi: 10.1038/s41413-024-00318-8 (PMC10909856; doi:10.1038/s41413-024-00318-8)
Supplement: Supplementary file 1 — Supplementary materials of "Targeted knockdown of PGAM5 in synovial macrophages efficiently alleviates osteoarthritis" [file 41413_2024_318_MOESM1_ESM.docx]

**Supplementary Materials**


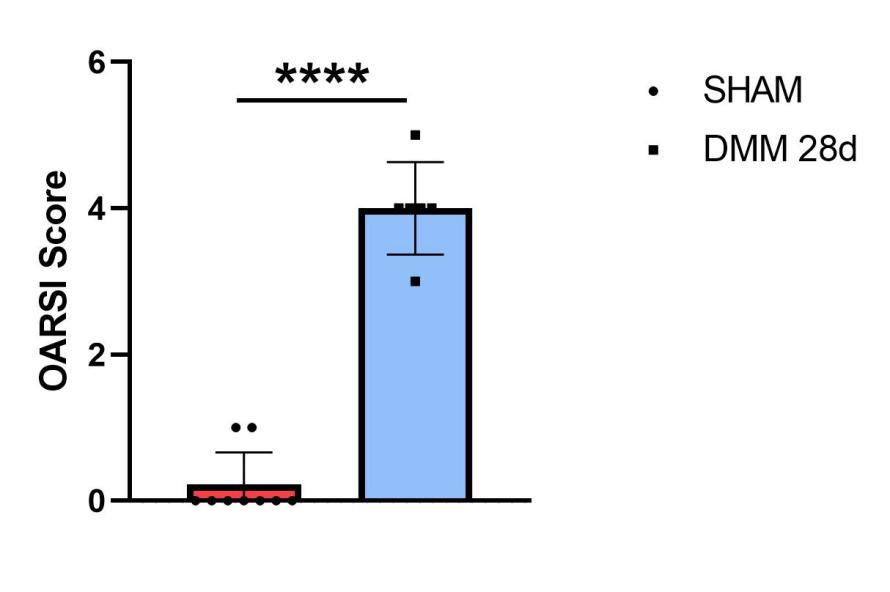


**Fig S1**. OARSI scores of safranin O staining were quantified in sham group and DMM group of WT mice in Figure 2d. Data are shown as the mean±s.d. *P<0.05, **P<0.01, ***P<0.001, ****P<0.0001 between the indicated groups. P values were determined using Student's t-tests.


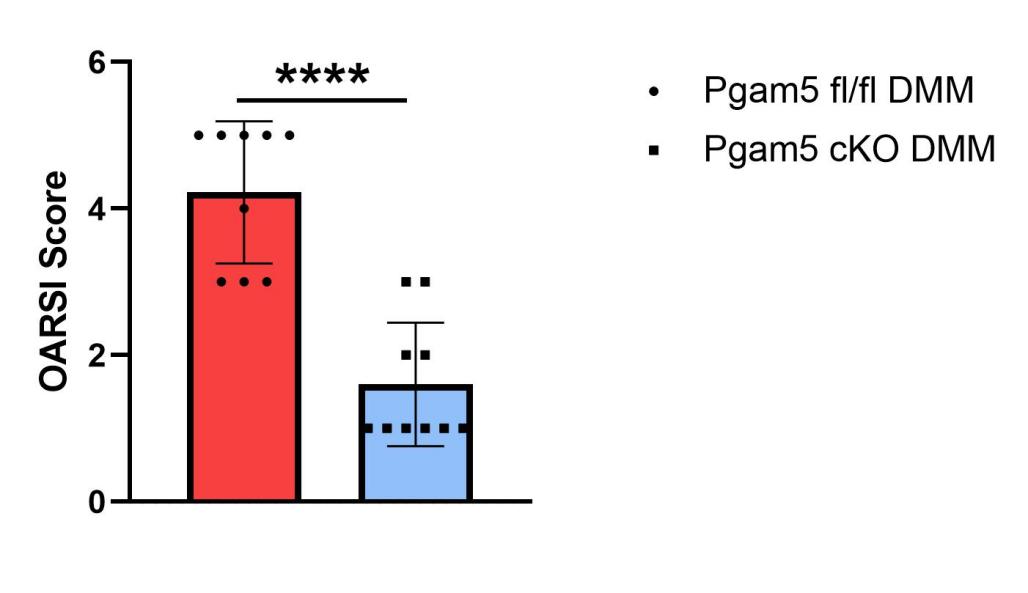


**Fig S2**. OARSI scores of safranin O staining were quantified in Pgam5 fl/fl DMM group and Pgam5 cKO DMM group of mice in Figure 3a. Data are shown as the mean±s.d. *P<0.05, **P<0.01, ***P<0.001, ****P<0.0001 between the indicated groups. P values were determined using Student's t-tests.


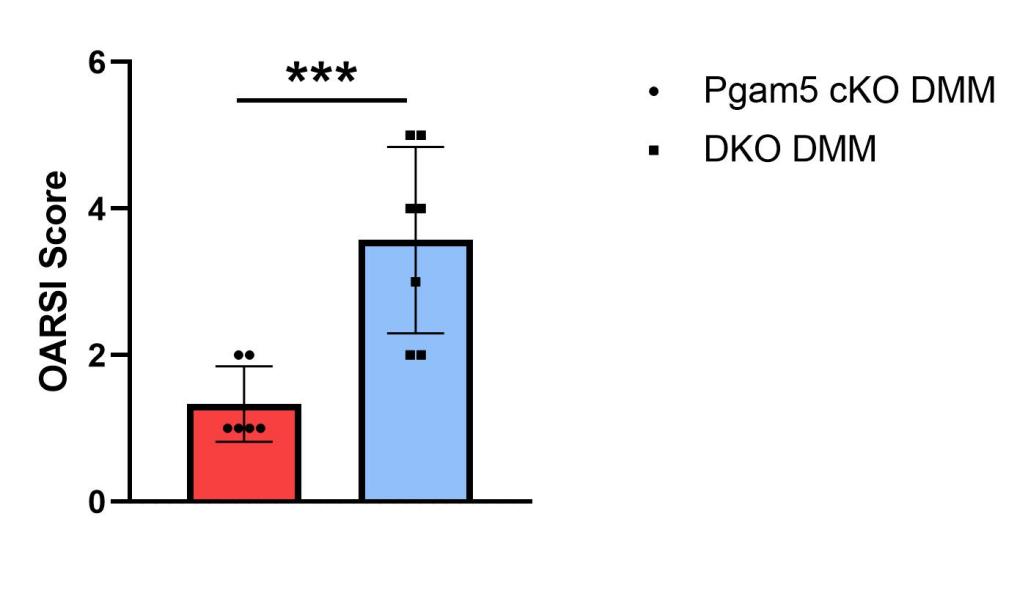


**Fig S3**. OARSI scores of safranin O staining were quantified in Pgam5 cKO DMM group and DKO DMM group of mice in Figure 6f. Data are shown as the mean±s.d. *P<0.05, **P<0.01, ***P<0.001 between the indicated groups. P values were determined using Student's t-tests.


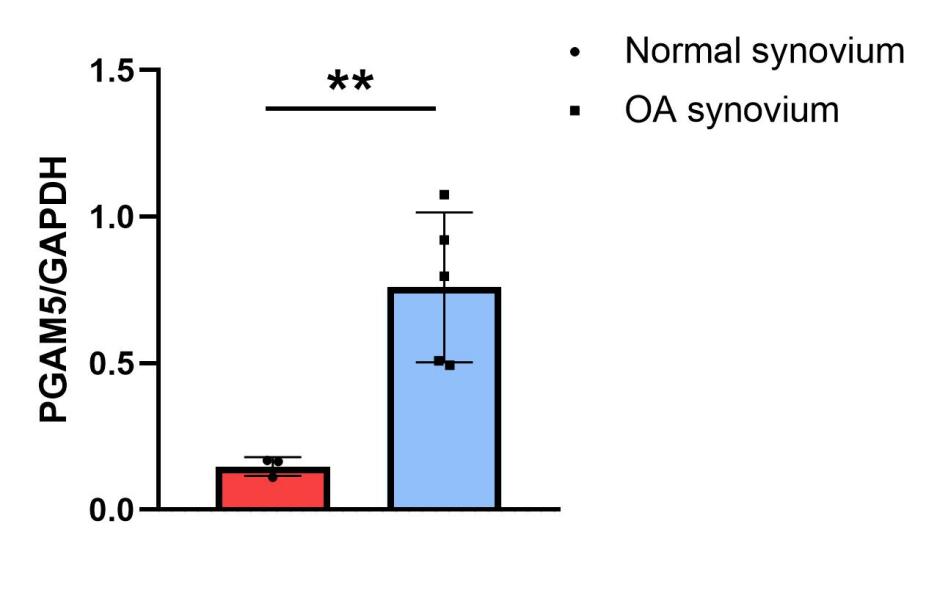


**Fig S4.** Relative protein levels of PGAM5 in human normal synovium and OA synovium. *P<0.05, **P<0.01 between the indicated groups. P values were determined using Student's t-tests.


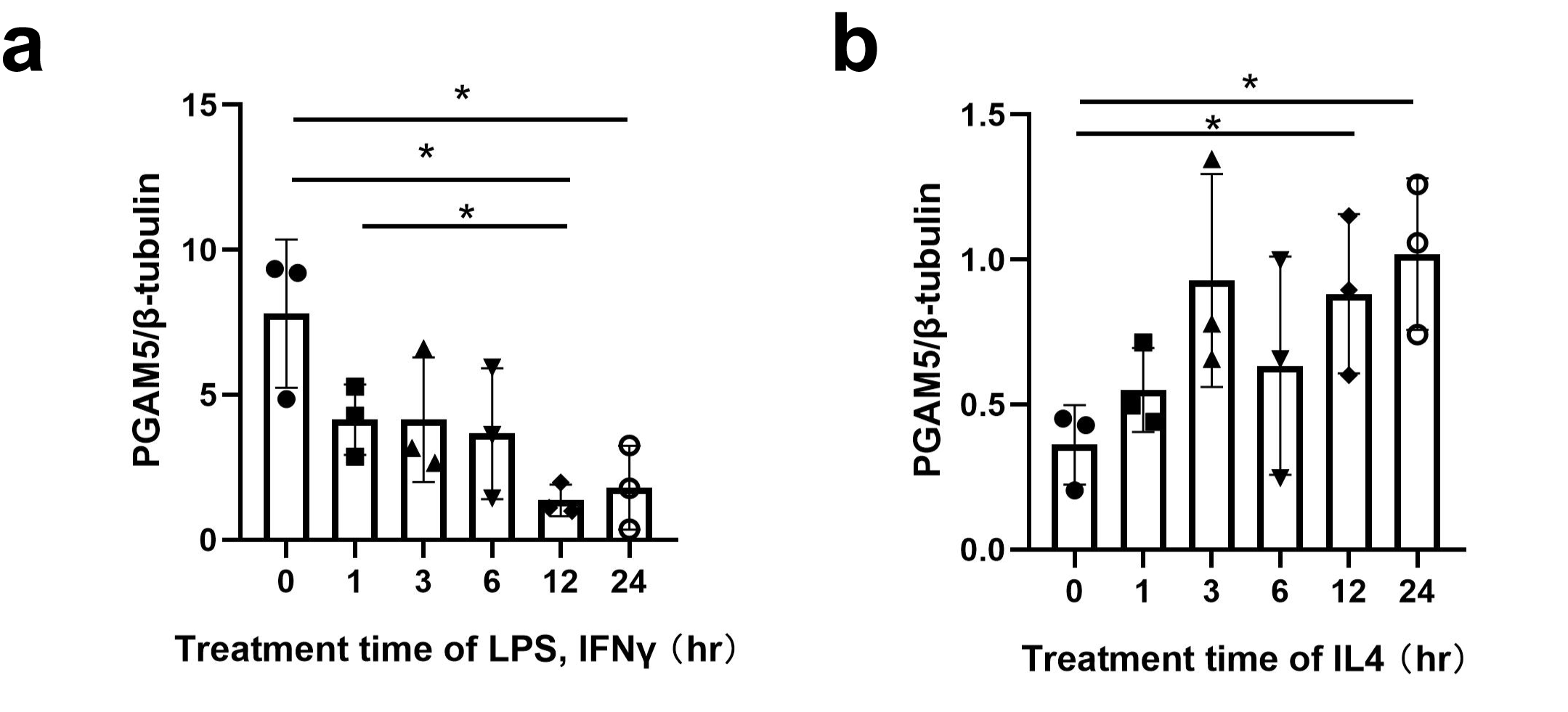


**Fig S5.** (a) Relative protein levels of PGAM5 of macrophages after treatment with LPS and IFNγ for 0, 1, 3, 6, 12, 24 hours. (b) Relative protein levels of PGAM5 of macrophages after treatment with IL4 for 0, 1, 3, 6, 12, 24 hours. *P<0.05 between the indicated groups. P values were determined using Student's t-tests.


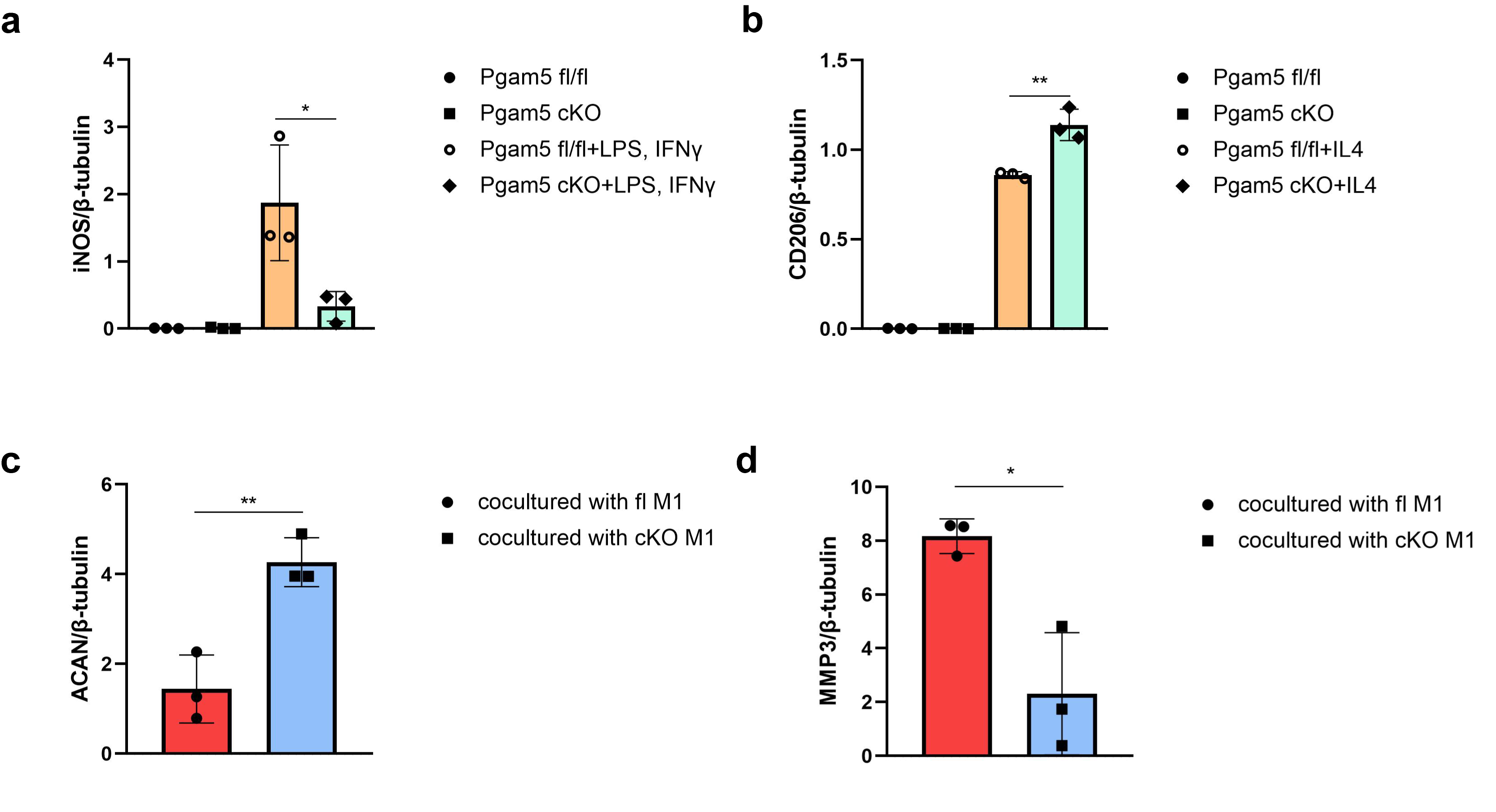


**Fig S6.** (a) Relative protein levels of iNOS of Pgam5 fl/fl and Pgam5 cKO macrophages treated with LPS and IFNγ. (b) Relative protein levels of CD206 of Pgam5 fl/fl and Pgam5 cKO macrophages treated with IL4. (c) Relative protein levels of ACAN of chondrocytes cocultured with Pgam5 fl/fl M1 and Pgam5 cKO M1 macrophages. (d) Relative protein levels of MMP3 of chondrocytes cocultured with Pgam5 fl/fl M1 and Pgam5 cKO M1 macrophages. *P<0.05, **P<0.01 between the indicated groups. P values were determined using Student's t-tests.


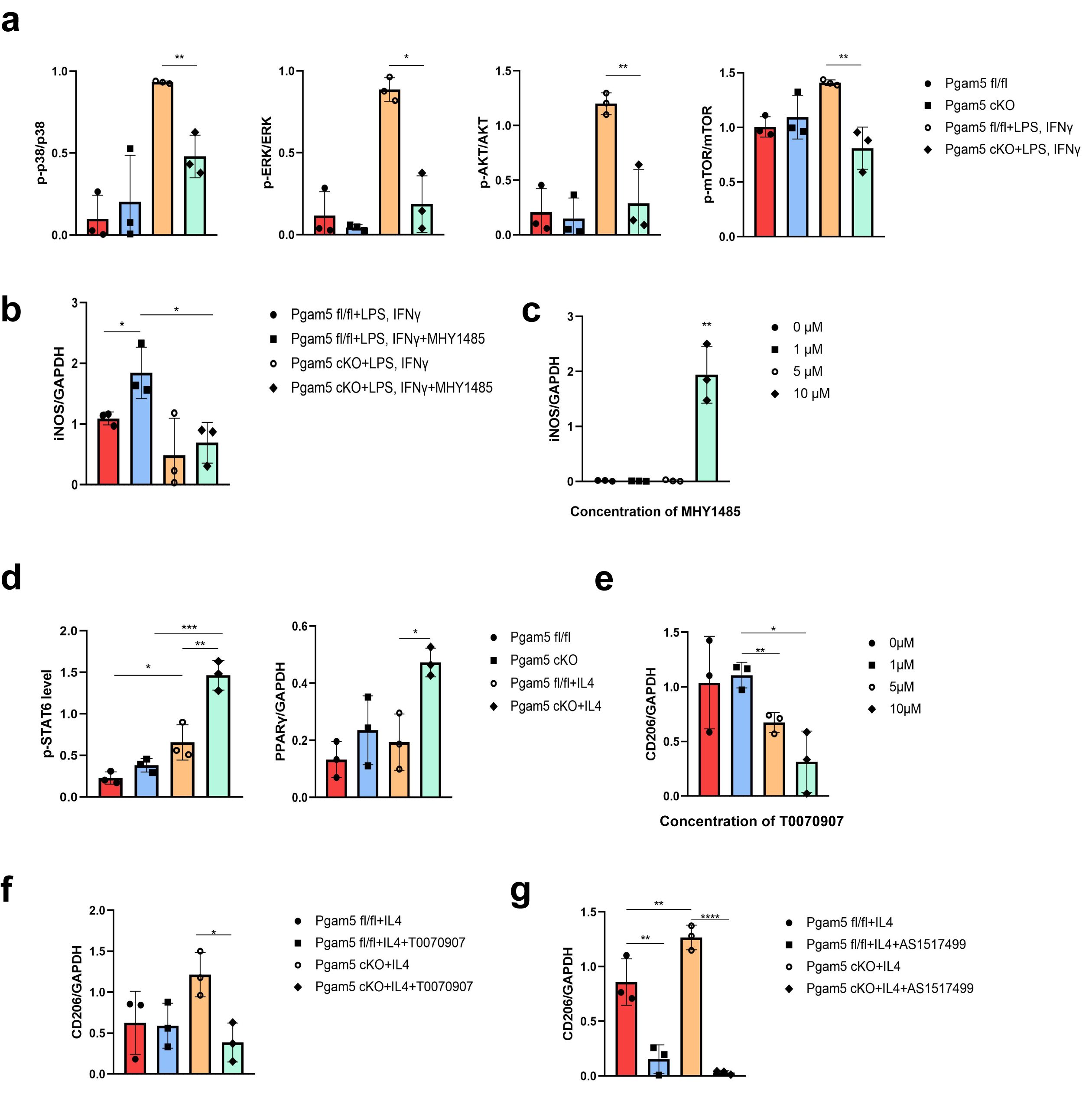


**Fig S7.** (a) Relative protein levels of p-p38, p-ERK, p-AKT, and p-mTOR of Pgam5 fl/fl and Pgam5 cKO macrophages treated with LPS and IFNγ. (b) Relative protein levels of iNOS of Pgam5 fl/fl and Pgam5 cKO macrophages treated with LPS, IFNγ, and MHY1485. (c) Relative protein levels of iNOS of Pgam5 cKO macrophages treated with 0, 1, 5, 10μM MHY1485. (d) Relative protein levels of p-STAT6 and PPARγ of Pgam5 fl/fl and Pgam5 cKO macrophages treated with IL4. (e) Relative protein levels of CD206 of Pgam5 cKO macrophages treated with 0, 1, 5, 10μM T0070907. (f) Relative protein levels of CD206 of Pgam5 fl/fl and Pgam5 cKO macrophages treated with IL4 and T0070907. (g) Relative protein levels of CD206 of Pgam5 fl/fl and Pgam5 cKO macrophages treated with IL4 and AS1517499. *P<0.05, **P<0.01, ***P<0.001, ****P<0.0001 between the indicated groups. P values were determined using Student's t-tests.

.


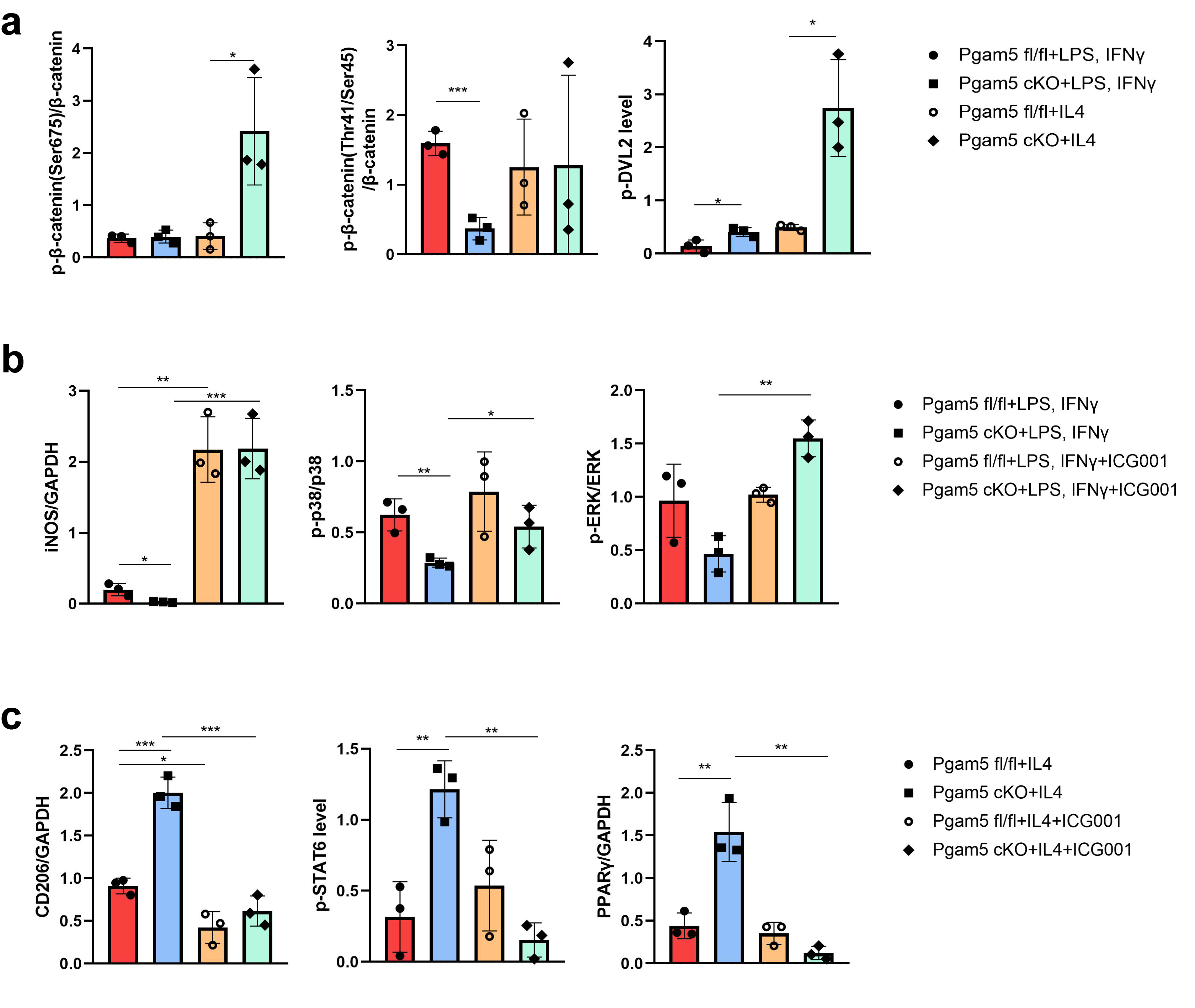


**Fig S8.** (a) Relative protein levels of p-β-catenin(Ser675), p-β-catenin(Thr41/Ser45), p-DVL2 of Pgam5 fl/fl and Pgam5 cKO macrophages treated with LPS, IFNγ or IL4. (b) Relative protein levels of iNOS, p-p38, p-ERK of Pgam5 fl/fl and Pgam5 cKO macrophages treated with LPS, IFNγ and ICG001. (c) Relative protein levels of CD206, p-STAT6, PPARγ of Pgam5 fl/fl and Pgam5 cKO macrophages treated with IL4 and ICG001. *P<0.05, **P<0.01, ***P<0.001 between the indicated groups. P values were determined using Student's t-tests.


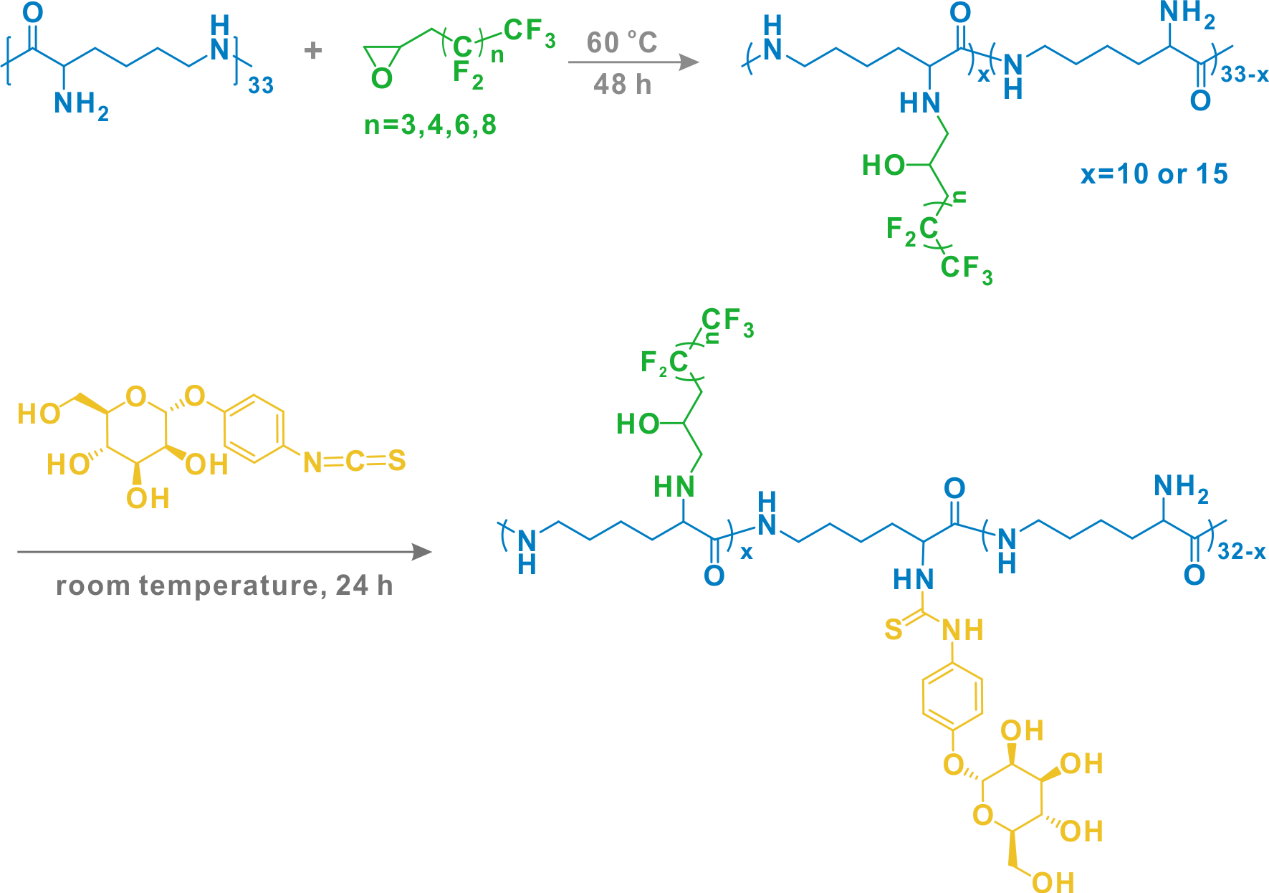


**Fig S9**. Synthesis of the mannose modified fluoropolymers.
